# Wastewater Surveillance for Norovirus, California, USA

**DOI:** 10.3201/eid3011.241001

**Published:** 2024-11

**Authors:** Alexander T. Yu, Elisabeth Burnor, Angela Rabe, Sarah Rutschmann, Marlene K. Wolfe, Jessie Burmester, Chao-Yang Pan, Alice Chen, Hugo Guevara, Christina Morales, Debra A. Wadford, Alexandria B. Boehm, Duc J. Vugia

**Affiliations:** California Department of Public Health, Richmond, California, USA (A.T. Yu, E. Burnor, A. Rabe, S. Rutschmann, C.-Y. Pan, A. Chen, H. Guevara, C. Morales, D.A. Wadford, D.J. Vugia); Emory University Rollins School of Public Health, Atlanta, Georgia, USA (M.K. Wolfe); County of San Luis Obispo Public Health Department, San Luis Obispo, California, USA (J. Burmester); Stanford University, Stanford, California, USA (A.B. Boehm)

**Keywords:** norovirus, wastewater surveillance, viruses, enteric infections, California, United States

## Abstract

Norovirus is a leading cause of acute gastroenteritis and imposes a substantial disease burden. In California, USA, norovirus surveillance is limited. We evaluated correlations between wastewater norovirus concentrations and available public health surveillance data. Wastewater surveillance for norovirus genotype GII in California provided timely, localized, and actionable data for public health authorities.

Norovirus infection causes substantial disease burden, but public health surveillance is limited, and cases are not routinely reported (*1*,*2*). Wastewater surveillance has the potential to provide localized data on norovirus transmission and outbreaks, which may improve public health awareness, communication, and prevention efforts. This study assessed whether wastewater-based norovirus surveillance data correlates with existing norovirus surveillance data and can improve the timeliness and representativeness of norovirus surveillance and inform public health action.

In 2022, the WastewaterSCAN program (https://www.wastewaterscan.org) began monitoring for norovirus genotype GII RNA in wastewater in California, USA, with the California Department of Public Health (CDPH) (*3**,**4*). We collected wastewater data during December 17, 2022–December 17, 2023, from 76 California wastewater utilities, including sites in all 5 California public health officer regions (*4*,*5*). We extracted viral RNA from wastewater settled solids and quantified norovirus concentrations by using digital droplet reverse transcription PCR (*5*). We normalized norovirus wastewater concentrations from individual sewersheds to pepper mild mottle virus (an internal recovery and fecal strength control), population-weighted them, and combined them into 5 California public health officer regional aggregates and a state aggregate (*4*,*5*).

We compared wastewater norovirus data to Centers for Disease Control and Prevention National Respiratory and Enteric Virus Surveillance System (NREVSS) norovirus test positivity at the national and western US regional level and to monthly California Norovirus Laboratory Network (NLN)–confirmed GII norovirus outbreaks. NREVSS receives norovirus test results from outbreaks or sporadic community cases from select participating laboratories (*2*,*6*). We did not analyze California-specific NREVSS test positivity data because of a paucity of data (average total reported monthly specimens <10). NLN tracks laboratory-confirmed norovirus outbreaks (>2 confirmed, epidemiologically linked cases). We compared 10-day center-aligned moving averages of wastewater aggregates (a wastewater averaging window routinely used at CDPH) to NREVSS test positivity data, which are reported as 21-day center-aligned moving averages. We summed NLN outbreaks over 30 days (because of low numbers of reported outbreaks) and compared them to 30-day averages of wastewater aggregates. We used Kendall rank correlation, a nonparametric test measuring the strength of dependence between 2 variables, for comparison because it is robust to small samples sizes and skewed data (*7*,*8*). We defined strong correlations as τ values >0.49 (*9*). We performed statistical analyses in R version 4.0.4 (The R Project for Statistical Computing, https://www.r-project.org).

We observed positive, statistically significant (p<0.001), moderate-to-strong correlations between California regional and statewide wastewater aggregates and US national and western regional NREVSS test positivity (median τ value 0.65 [range 0.46–0.77]). We also observed positive, statistically significant (p<0.01), moderate-to-strong correlations between California wastewater aggregates and monthly California norovirus outbreaks (median τ value 0.65 [range 0.57–0.73]) (Table). We observed the lowest correlations for the Rural Northern California region, possibly because that region has the lowest wastewater surveillance population coverage, a largely rural population, and no NREVSS reporting laboratory. The lack of NREVSS reporting laboratories suggests that local norovirus activity may not be represented in western US regional- or national-level surveillance, highlighting the potential value of wastewater surveillance to provide localized information.

**Table:** Kendall correlations between 10-day center-aligned rolling averages of regional wastewater aggregated data and 21-day center-aligned averages of NREVSS test positivity (NREVSS analysis) and 30-day averages of regional wastewater aggregated data and 30-day counts of statewide norovirus outbreaks (NLN outbreak analysis), California, USA, December 17, 2022–December 17, 2023*

Wastewater region	NREVSS analysis				NLN outbreak analysis	
	NREVSS region	Kendall τ	p value		Kendall τ	p value
State	National	0.754	<0.001		0.701	0.002
State	Western region	0.682	<0.001			
Bay Area	National	0.770	<0.001		0.734	0.001
Bay Area	Western region	0.751	<0.001			
Greater Sacramento	National	0.644	<0.001		0.734	0.001
Greater Sacramento	Western region	0.666	<0.001			
Rural Northern California	National	0.464	<0.001		0.571	0.01
Rural Northern California	Western region	0.458	<0.001			
San Joaquin Valley	National	0.487	<0.001		0.603	0.01
San Joaquin Valley	Western region	0.641	<0.001			
Southern California	National	0.654	<0.001		0.603	0.002
Southern California	Western region	0.564	<0.001			

*NLN, California Norovirus Laboratory Network; NREVSS, Centers for Disease Control and Prevention National Respiratory and Enteric Virus Surveillance System.

Wastewater norovirus data suggested distinct regional and temporal patterns of norovirus activity within California, peaking as early as February 22, 2023, in Southern California and as late as March 24, 2023, in the San Joaquin Valley (Figure). Those regional patterns were not discernable from NLN or NREVSS data. NLN outbreak data suggested that norovirus outbreaks peaked in March 2023 (Appendix Figure), whereas NREVSS test positivity peaked nationally on March 18, 2023, and in the western US region on April 22, 2023 (Figure).

**Figure:**
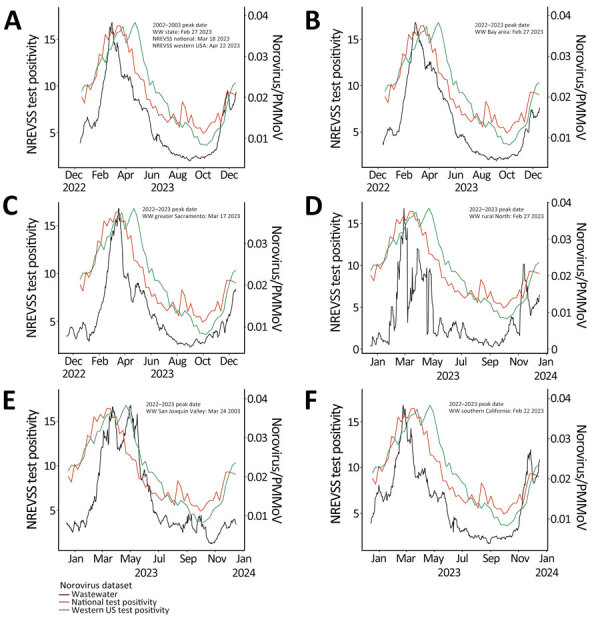
NREVSS norovirus test positivity (21-day center-aligned moving average) nationally (orange lines) and for the western United States (green lines) and wastewater aggregates (10-day center-aligned moving average) for norovirus, normalized by PMMoV (black lines), California, USA, December 17, 2022–December 17, 2023. A) Statewide; B) Bay Area; C) greater Sacramento; D) rural northern California; E) San Joaquin Valley; F) southern California. NREVSS, Centers for Disease Control and Prevention National Respiratory and Enteric Virus Surveillance System; PMMoV, pepper mild mottle virus; WW, wastewater.

Existing norovirus surveillance is limited and lacks widespread testing and reporting to public health authorities. California surveillance relies on successful outbreak investigations. Weekly California outbreak counts reported by NLN during 2022–2023 were small (median 0 [range 0–8]), which may represent a fraction of the actual number of outbreaks.

Wastewater results are available within 24–48 hours of sample collection and summarized into regular reports distributed to CDPH teams and local health departments (LHDs). In response to sustained wastewater norovirus increases, CDPH has issued California Health Alert Network notifications and Communicable Disease Briefs alerting LHDs of increasing norovirus activity and outbreak potential. Given that no other local California norovirus surveillance data are available, wastewater data have been used as a local and leading indicator to support investigations of gastrointestinal illness outbreaks. Those data have enabled LHDs to more (or less) aggressively pursue investigation and control efforts during gastroenteritis outbreaks, efforts that are time- and resource-intensive for LHDs, the public, and affected establishments. Further statistical analyses exploring lag times between wastewater concentrations and norovirus surveillance data and investigations into how different wastewater data smoothing and aggregation methods affect correlations will provide further insight into interpreting wastewater concentrations.

In conclusion, wastewater norovirus GII data from California during 2022–2023 correlated well with existing public health surveillance data. The wastewater data provided otherwise unavailable situational awareness, enabled timely identification of distinct California regional norovirus trends, and led to direct public health action, including guiding local outbreak investigations.

Appendix Additional information about wastewater surveillance for norovirus, California, USA.
