# Supplementary material for: Wastewater Surveillance for Norovirus, California, USA
Source: Emerg Infect Dis. 2024 Nov;30(11):2438–41. doi: 10.3201/eid3011.241001 (PMC11521154; doi:10.3201/eid3011.241001)
Supplement: Appendix — Additional information about wastewater surveillance for norovirus, California, USA. [file 24-1001-Techapp-s1.pdf]

Article DOI: https://doi.org/10.3201/eid3011.241001

EID cannot ensure accessibility for supplementary materials supplied by authors. Readers who have difficulty accessing supplementary content should contact the authors for assistance.

Wastewater Surveillance for Norovirus, California, USA

Appendix

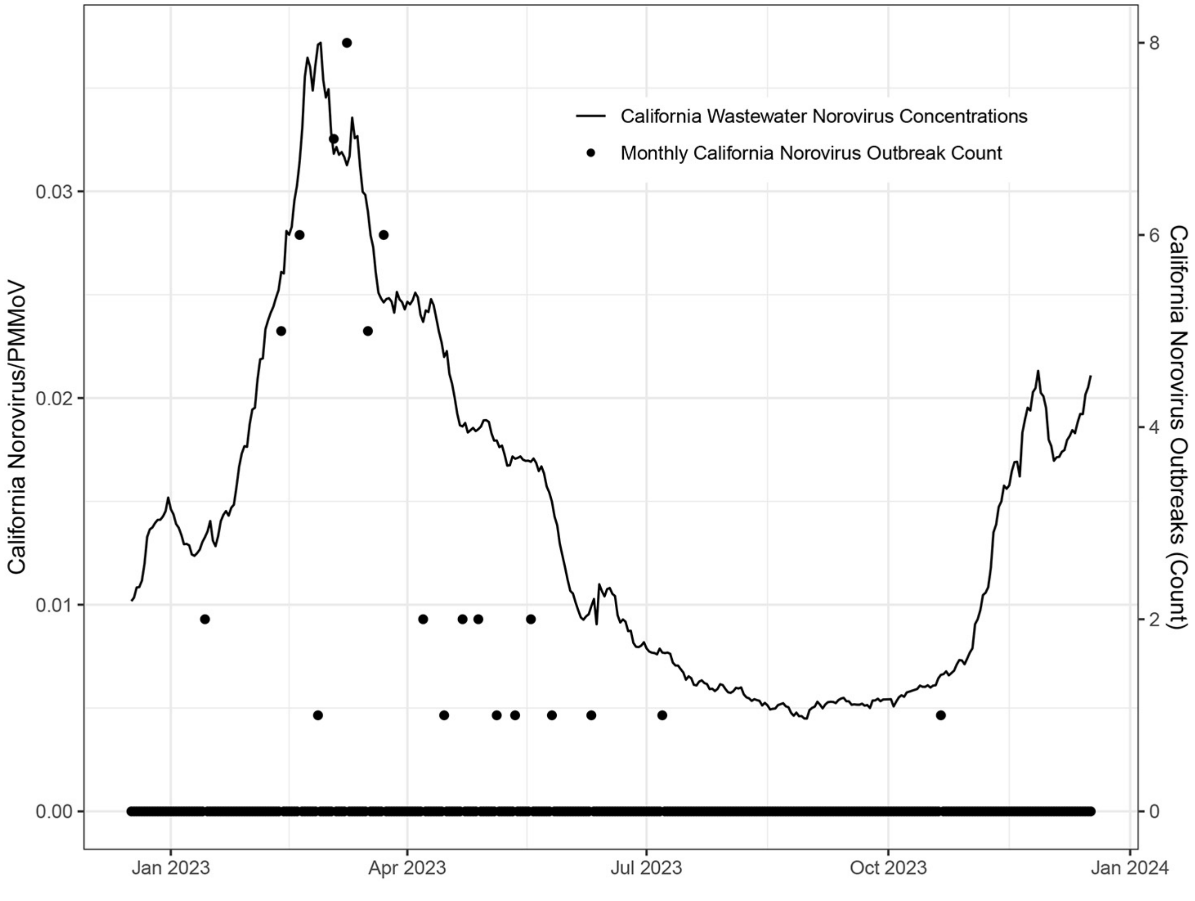

Appendix Figure. California norovirus outbreak monthly count and California state wastewater norovirus concentrations (10−day mean).
